# The Quality of Childbirth in The Light of Research the New Guidelines of The World Health Organization and Polish Perinatal Care Standards

**DOI:** 10.34763/devperiodmed.20192301.5459

**Published:** 2019-04-08

**Authors:** Barbara Baranowska

**Affiliations:** 1 Department of Midwifery, Centre of Postgraduate Medical Education, Warsaw, Poland

**Keywords:** perinatal care, quality of care, World Health Organization, opieka okołoporodowa, jakość opieki, Światowa Organizacja Zdrowia

## Abstract

The quality of birth is assessed by means of a comprehensive approach to the process of coming in to the world, taking into account the perspective of the mother and the child and the influence of labour on their future health and life. According to the recommendations of the World Health Organization, the delivery of every child should be consistent with the mother's personal and socio-cultural beliefs and should meet her expectations as to the care provided.

## Streszczenie

Jakość narodzin stanowi całościowe ujęcie procesu przychodzenia na świat uwzględniające perspektywę matki i dziecka oraz wpływ porodu na ich dalsze zdrowie i życie. Według zaleceń Światowej Organizacji Zdrowia, poród każdej kobiety powinien być zgodny z jej osobistymi i społeczno-kulturowymi przekonaniami, a także odpowiadać jej oczekiwaniom co do świadczonej opieki.

## Introduction

The way in which we come into the world determines aur future life. As F. Leboyer, an activist for humanized obstetrics wrote, "Birth can be a moment, but it is an exceptional moment" [1]. The last several decades provide evidence for the importance of the perinatal period for the health of an individual and for society. Epigenetics changed the perception of health as determined by inherited traits. Environmental impact and metabolic programming are becoming new elements in the consideration of improving the quality of perinatal care. For the mother and her family the act of giving birth is one of the most important and often most difficult life experiences.

Currently we are observing a departure from the perception of childbirth as a purely medical phenomenon, evaluated only with the help of clinical indicators, either obstetric or neonatal. Tuus, one can speak about the quality of birth - where birth is approached from the bio-psycho-social perspective of both the mother, the child, and their future health, taking into account the quality of care provided, the woman’s expectations of care and her experience of childbirth. Reducing perinatal mortality of women and children, especially in developed countries, is not the only goal in the area of improving women’s life and health. The way in which the mother and her family experience birth is increasingly often taken into account as a factor influencing health policy on reproductive health.

## Research on the quality of delivery and perinatal care

The need to consolidate activities for better perinatal care serving the health of both mother and child was expressed by the work of the team of the European Cooperation in Science and Technology (COST) Action Birth [2]. Researchers and specialists from various areas have joined forces to study human birth. Over 100 scientists from 30 countries have been involved in the project: architects, psychologists, biologists, anthropologists and artists.

As part of the project, research was planned and carried out in the fields of:

- Biomedicine – issues related to epigenetics in relation to the perinatal period and the resulting long-term health consequences;
- Biomechanics – problems of mechanics and bioengineering in relation to pregnancy and childbirth;
- Socio-cultural perspectives – childbirth understood as a socio-cultural phenomenon, issues of dissonance between the prevailing cultural social expectations and marginalized groups, such as immigrant women or LGBT persons; Lesbian, Gay, Bisexual, Transgender);
- Organizational perspectives – issues of health care organization, economic costs related to childbirth interventions;
- Neuro-psycho-social perspectives – observational studies of neuro-psycho-social traits related to the birth event, with particular emphasis on the impact of personal interactions and behaviour of staff providing care to women, the relationship between staff and women and their families. Study of the impact of these relationships on the neurohormonal course of delivery.

Currently work is under way on analysing and dissemination of research results that have been obtained in the course of the international project, and are to serve scientists, clinicians, managers and politicians in creating new conditions for a dignified and satisfying birth.

A lot of space has been devoted to epigenetic aspects. Mechanisms influencing the change in gene expression associated with the way or course of labour have an impact on the health of newborns and their mothers (tab. I). Modification of the genetic baggage, which is hereditary from generation to generation, occurs already during pregnancy and birth, affecting the increase or reduction of the risk of future diseases [3, 4, 5, 6, 7]. Therefore, the assessment of the quality of birth should also be considered in the context of long-term effects. The importance of epigenetic mechanisms and factors shaping the human microbiome should be taken into account when planning perinatal care [5].

**Table I:** Examples of the impact of childbirth on the life of mother and child (own study). Tabela I. Przykłady wpływu przebiegu porodu na życie matki i dziecka (opracowanie własne).

Increased risk of diseases in children born surgically, such as asthma, allergy, obesity, inflammatory diseases of the intestines and stomach mucosa, diabetes, irritable *Wzrost ryzyka* bowel *chorób* syndrome, *u dzieci* or leukaemia. *urodzonych drogą operacyjną, takich jak astma, alergia, otyłość, choroby zapalne jelit i błon śluzowych żołądka, cukrzyca, zespół jelita drażliwego, czy białaczka*.	[3, 8, 9, 10, 11, 12, 13, 14, 15, 16]
Worse results, in the first few months after delivery, in terms of physical, mental, social and pain indicators related to health-related quality of life, as well as lower energy levels and vitality in women who gave birth through Caesarean section. *Gorsze wyniki, w ciągu pierwszych kilku miesięcy po porodzie, w zakresie wskaźników fizycznych, umysłowych, społecznych i bólowych jakości życia związanych ze zdrowiem, a także niższy poziom energii i witalność u kobiety, które rodziły poprzez cięcie cesarskie*.	[17]
Higher risk of postnatal depression and somatic symptoms, affecting the creation of bonds between mother and child in a woman who during the delivery received *Wyższe ryzyko* exogenous *wystąpienia* oxytocin. *depresji poporodowej i objawów somatyzacyjnych, mającej wpływ na tworzenie więzi między matka i dzieckiem u kobiety, które w trakcie porodu otrzymały egzogenną oksytocynę*.	[18, 19]

## Evaluation of the quality of perinatal care – Polish and world standards

The definition of the assessment of the quality of care used by the World Health Organization speaks of the extent to which health services offered to individuals and populations improve the desired health outcomes [20]. High quality care should be safe, effective (based on scientific knowledge and guidelines), timely, efficient, equitable and patient [20].

Care for a pregnant woman and woman giving birth can be seen from two perspectives as: **the quality of care provided and the quality of care perceived through the woman’s personal experience**. The first, so called ‘technocratic’ perspective, focuses primarily on achieving certain standards by care providers (including health care workers) [21, 22]. The second assumes that the experience of the woman and her family related to the birth of a child is an important element in defining and evaluating the quality of healthcare [23, 24].

The structure of the quality of care for the mother and child proposed by WHO distinguishes female care experience as an equal pillar of quality (tab. II) [25].

**Table II:** The quality of care structure proposed by the World Health Organization [25] (own modification). Tabela II. Struktura jakości opieki proponowana przez Światowq Organizację Zdrowia (modyfikacja własna).

Quality of care *Jakość opieki*	
Provision of care *Świadczenie opieki*	Experience of care *Doświadczenie opieki*
1 – Evidence-based practices for routine care and management of complications *Oparta na dowodach opieka w sytuacjach standardowych i w przypadku wystąpienia komplikacjach*	4– Effective communication *Efektywna komunikacja*
2 – Actionable information systems *Działający system informacyjny*	5 – Respect and dignity *Szacunek i godność*
3 – Functional referral system *Funkcjonalny system referencyjności*	6– Emotional suport *Wsparcie emocjonalne*
7 – Competent and motivated human resources *Kompetentne i zmotywowane zasoby ludzkie*	
8– Essential facilities available *Niezbędne zasoby fizyczne*	

The latest WHO guidelines, „Intrapartum care for a positive childbirth experience” emphasizes the need to take into account the expectations of women who, in addition to being ensured the treatment consistent with evidence-based medicine, influence the building of a positive labour experience [26]. The set of rules regarding perinatal care has been extended by 26 recommendations. What was emphasized was the message concerning the provision of an individual approach to each woman giving birth, respectful care and effective communication between medical personnel and women. Experts from the World Health Organization asked themselves: what do women want, what do they need and what do they value in the process of giving birth? Analysing the results of a systematic review based on qualitative research, they found that the most important value for mothers is the positive experience of labour.

A negative childbirth experience influences the procreative decisions of women, their relationship with the child and the quality of their lives. [27].

The delivery experience is influenced by many factors [28]. They can be enumerated as dependent on:

- the manner of care provided (including elements of equipment and facilities, the technical and communication skills of staff, the compliance of the procedures taken with evidence-based medicine),
- the personal qualities of a woman (including the degree of preparation for delivery, the ability to cope during delivery and the attitude to the child),
- stress experienced during birth (most often associated with obstetric injuries, medical interventions, severe anxiety, inability to cope with pain, long delivery, the poor condition of the child following birth).

Polish standards of perinatal care emphasize the subjectivity of the woman giving birth and the importance of assessing the quality of care provided. The Ordinance of the Minister obliges medical facilities providing perinatal care to “establish indicators of perinatal care, guided in particular by (...) the assessment of the satisfaction of women in care” [29].

**Quality of childbirth – a comprehensive approach** Existing methodologies and tools supporting the operationalisation of the key features of the quality of maternity care do indeed fail to consider the overall perception of the birth process, taking into account the long-term perspective. An example of this is the tool proposed by the World Health Organization “Hospital care for mothers and newborn babies quality assessment and improvement tool” or “The maternal-newborn bottleneck analysis tool” [30, 31, 32, 33].

The term quality of childbirth refers to the overall perception of the process of coming into the world, taking into account both the quality of maternity care, the experience of a woman, the child and the whole family, as well as short and long-term health effects for the mother and child [17, 34, 35].

**Fig. 1:**
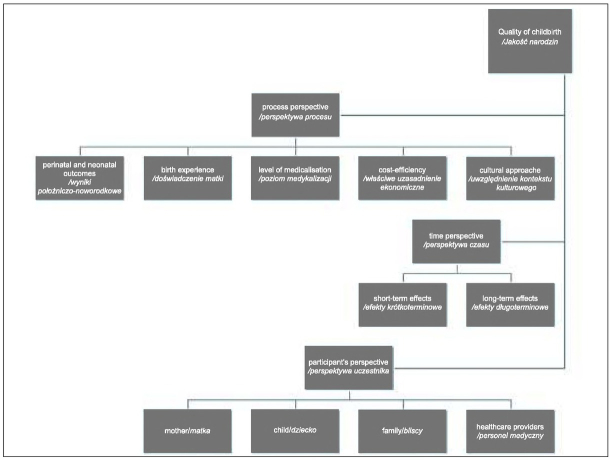
Quality of birth in terms of three perspectives (own study). Ryc. 1. Jakość narodzin w ujęciu trzech perspektyw (opracowanie własne).

Therefore, all the perspectives must be taken into account in assessing the quality of birth, those dealing with the process itself, but also with time and individual participants (Fig. 1).

When a woman decides to have a caesarian section on demand, without indications, and gives birth to a healthy child who from the first day of life is fed with a baby formula, despite the objective indicators of a successful operation, the mother’s satisfaction and Apgar scoring testifying to the child’s good condition does not give a full picture of the quality of birth. In epigenetic terms, the lack of colonization by the mother’s microflora, prophylactic antibiotic therapy, the lack of the protective action of the colostrum, are a threat to the child, due to the lack of proper support of the immune system, and thus increases the risk of diabetes, leukaemia or obesity in adulthood.

As M. Odent wrote: “To change the world, we must first change the way the babies are being born” [36]. Every person providing care to a woman giving birth and her child takes on the responsibility not only for their lives, but for the quality of birth. The perception of coming into the world that takes into account all the perspectives and constant quality assessment gives new opportunities for planning and carrying out perinatal care that takes full account of the quality of birth. [37, 38, 39, 40].
